# The multifunctional Staufen proteins: conserved roles from neurogenesis to synaptic plasticity

**DOI:** 10.1016/j.tins.2014.05.009

**Published:** 2014-09

**Authors:** Jacki E. Heraud-Farlow, Michael A. Kiebler

**Affiliations:** 1 Department of Chromosome Biology, Max F. Perutz Laboratories, University of Vienna, 1030 Vienna, Austria; 2 Department of Anatomy and Cell Biology, Ludwig-Maximilians-University, 80336 Munich, Germany

**Keywords:** RNA localisation, Staufen, neurogenesis, synaptic plasticity, RNP, mRNA stability, learning and memory

## Abstract

Staufen (Stau) proteins belong to a family of RNA-binding proteins (RBPs) that are important for RNA localisation in many organisms. In this review we discuss recent findings on the conserved role played by Stau during both the early differentiation of neurons and in the synaptic plasticity of mature neurons. Recent molecular data suggest mechanisms for how Stau2 regulates mRNA localisation, mRNA stability, translation, and ribonucleoprotein (RNP) assembly. We offer a perspective on how this multifunctional RBP has been adopted to regulate mRNA localisation under several different cellular and developmental conditions.

## Highlights

• Staufen (Stau) proteins have evolutionarily conserved functions in the brain.
• Stau proteins asymmetrically segregate mRNAs during mouse and fly neurogenesis.
• Stau proteins regulate synaptic plasticity and memory formation in flies and mammals.
• Stau proteins have roles in translation, localisation, and ribonucleoprotein formation.
• New data indicate that mammalian Stau1 and Stau2 can both stabilise and destabilise target mRNAs.

## RNA localisation in the CNS

The localisation of RNA to distinct regions of the cell allows restricted protein synthesis, leading to spatially controlled adaptations within the cell. This is achieved through the actions of an ensemble of proteins (and probably regulatory ncRNAs) which direct the fate of mRNAs via nuclear export, mRNA stability, transport, and translational control [1]. This fundamentally important process can be envisaged to be performed by a dynamic molecular machine [2] that is utilised many times throughout development. In neurons, many RNAs are localised to both the axon and dendrites where their protein products modify the local compartment; for example, the growth cone during axon outgrowth or an individual synapse during memory formation [3]. Disruption of this process may have severe consequences because mutations in RBPs with known roles in local translation have been linked to several human neurologic diseases [4]. We discuss here new data pertaining to the function of Stau-containing RNPs during RNA localisation in the nervous system. In the past decade Stau proteins have emerged as crucial regulators of several aspects of neuron development and function (Box 1).

Box 1 **A short history of Stau proteins in the brain**

**Figure 1:**
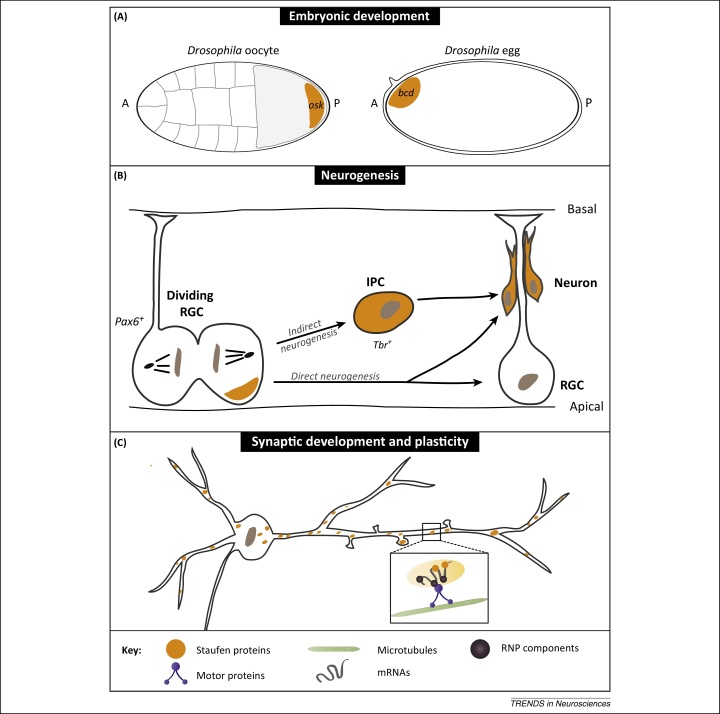
Staufen (Stau) proteins have conserved functions during three stages of development. **(A)** Stau proteins have a conserved role in early embryonic development (see main text). Depicted here is the localisation of *Drosophila* Stau in the oocyte and egg. Stau is crucial for the localisation of *oskar* (*osk*) mRNA to the posterior (P) pole of the oocyte (left side) and *bicoid* (*bcd*) mRNA to the anterior (A) pole of the egg (right side). The orange shading indicates the localisation of Stau protein. **(B)** During mouse neurogenesis, radial glial cells (RGCs; Pax6^+^) divide asymmetrically to produce another RGC and a post-mitotic neuron (‘direct neurogenesis’). In the case of ‘indirect neurogenesis’ the RGC produces another RGC and an intermediate progenitor cell (IPC; Tbr^+^), which divides once more symmetrically to produce two neurons. During maturation, the neurons migrate along the RGC fibre away from the apical surface towards the cortical plate (‘basal’). Stau2 protein (orange shading) localises into the differentiating cell (IPC or neuron), promoting differentiation and suppressing the stem cell state (see also Figure 2A). Stau is also important during *Drosophila* neurogenesis where it localises cell fate determinants into the differentiating ganglion mother cell (GMC). **(C)** Homologues of Stau in *Drosophila*, *Aplysia*, and rodents have functions in synaptic development and plasticity. The somatodendritic localisation of Stau2 protein (orange dots) is depicted here in a mature neuron. The protein forms RNPs with mRNAs and other proteins that traffic bidirectionally along microtubules in dendrites. Stau proteins are involved in memory formation and plasticity, and are believed to contribute to the transport and activity-dependent translation of localised mRNAs (see Figure 2B).

The Stau family of double-stranded RNA-binding proteins (dsRBPs) exhibit a conserved function in RNA localisation, which is supported by data in *Drosophila*, *Xenopus*, *Aplysia*, zebrafish, and mouse 18, 28, 69, 70, 73, 78 (see Figure 1 in main text). Stau was originally described for the localisation of mRNAs encoding cell fate determinants essential for the anterior–posterior patterning of the *Drosophila* oocyte (see Figure 1A in main text) 69, 79. However, mutants in *Drosophila stau* also affect brain development and function 13, 69, 80. During *Drosophila* neuroblast mitosis, Stau mediates the asymmetric localisation of *prospero (pros)* mRNA to the future ganglion mother cell (GMC), away from the neuroblast, thus promoting differentiation [81]. In the GMC, the Pros transcription factor is a crucial cell fate determinant that acts to promote GMC fate (differentiation) and suppress the stem cell fate [82]. The GMC then goes on to divide once more to produce two neurons. New data now indicate that this role in fly neurogenesis is conserved in the mammalian brain (see main text).

Early work from the laboratory of Tully then showed *Drosophila* Stau is also important in the adult brain. One-day memory is abolished when temperature sensitive *stau* mutants are shifted to the non-permissive temperature immediately after training that induces long-term memory (LTM), suggesting an early role for *stau* in LTM formation [13]. In addition, Stau is required for long-term facilitation of *Aplysia* motor neurons in response to serotonin, indicating that roles in plasticity are not limited to flies [78].

Mammals express two orthologues of Stau, Stau1 and Stau2, and both exist in several splice isoforms 49, 83, 84. Stau1 is expressed in most cell types, including neurons, whereas Stau2 is enriched in the brain and only expressed at low levels in other tissues. The two proteins are predominantly found in distinct particles in dendrites of primary rodent hippocampal neurons, suggesting they may have distinct functions (see Figure 1C in main text) [84]. Studies of Stau1- and Stau2-deficient neurons now also support this view (see main text). In the first functional studies in mammalian hippocampal neurons, knockdown of Stau2 resulted in fewer, extended dendritic spines compared to controls, which corresponds to a reduction in the number of synapses [18]. These changes also lead to a reduction in miniature excitatory postsynaptic current (mEPSC) amplitudes, indicating a defect in synaptic transmission through postsynaptic glutamate receptors. Stau1 is also important for mammalian neuronal morphogenesis and plasticity, although in apparently non-redundant pathways to Stau2 19, 21, 22.

Recent studies in both *Drosophila* and mammalian neurons have now extended these early studies, uncovering roles of Stau proteins in dendrite morphogenesis, plasticity, and memory formation. The mRNAs and molecules that underlie the adult brain Stau phenotypes are beginning to be uncovered, and these suggest that Stau1 and Stau2 are not only involved in RNA transport but also in mRNA stability and translation (see main text).

## Stau-mediated asymmetric cell division during neurogenesis

During cell division, cellular components are distributed equally between daughter cells to ensure faithful replication and expansion of the given cell type. In specialised cases, however, asymmetric distribution is used to generate daughter cells with different cell fates [5]. The division of the *Drosophila* neuroblast during neurogenesis has served as an ideal model system in which to study asymmetric cell division. It was during this process that the role of Stau in neurogenesis was first uncovered (Box 1).

Until recently, however, the role of Stau proteins in mammalian neurogenesis had not been investigated. Two new papers now show that Stau2 makes a crucial contribution to cell fate specification during neurogenesis in mice 6, 7. Neurogenesis commences at around embryonic day 13 (E13), when asymmetric cell division of radial glial cells (RGCs) begins, producing another RGC and either an intermediate progenitor cell (IPC) or a post-mitotic neuron (Figure 1B). RGCs are the founder cells for a large proportion of the neurogenic lineages in the CNS and thus are of fundamental importance to brain development [8]. At this time, Stau2 starts to polarise in mitotic cells both *in vitro* and *in vivo* where it localises to the differentiating cell [7]. This is consistent with *Drosophila* Stau, which also segregates into the differentiating GMC.

Short hairpin RNA (shRNA)-mediated knockdown of Stau2 both *in vitro* and *in vivo* results in an ∼50% decrease in the number of Pax6 (paired box 6)-positive RGCs, and a concomitant two- to threefold increase in the number of more differentiated daughter cells 6, 7. This is therefore consistent with premature neuronal differentiation in the absence of Stau2. Associated with an increase in the number of neurons is defective migration of the excess neurons to the cortical plate. It is unclear, however, whether this is the result of an intrinsic defect in migration of the neurons or the loss of their scaffold – which is normally provided by the long fibres of the now depleted RGCs (Figure 1B).

**Figure 2:**
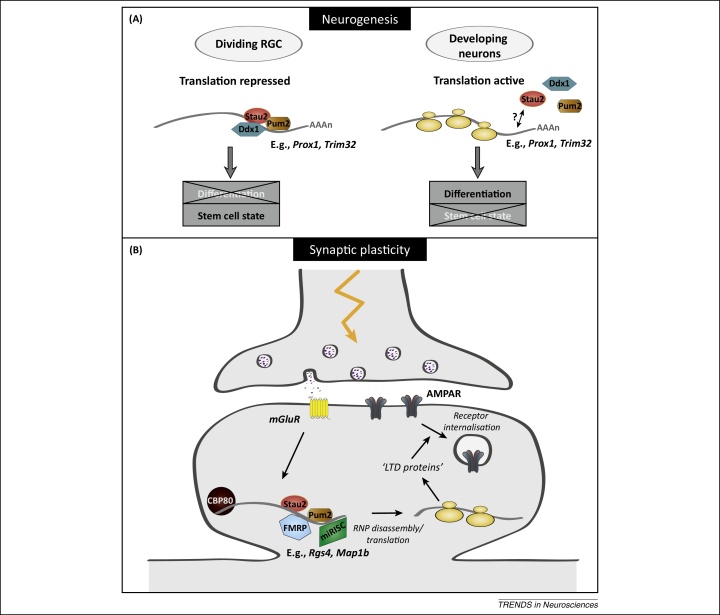
Molecular models for the role of Staufen 2 (Stau2) during neurogenesis and in synaptic plasticity. Two models are presented for Stau2 function in (A) developing and (B) mature neurons. Some components of the ribonucleoproteins (RNPs) may be shared between the two developmental processes (e.g., Pum2, pumilio RNA-binding family member 2), whereas others are specific [e.g., Ddx1, DEAD (Asp-Glu-Ala-Asp) box helicase 1]. Likewise, there may be some mRNA targets that are common across development (e.g., *Rgs4*, regulator of G-protein signaling 4), whereas others are temporally and spatially specific. **(A)** Model of Stau2 RNP function during neurogenesis. Stau2 forms RNPs in dividing radial glial cells (RGCs) with the RBPs, Ddx1 and Pum2, as well as with mRNAs, and these segregate into the differentiating daughter cell [intermediate progenitor cell (IPC) or neuron; Figure 1]. mRNAs found in these RNPs include *Prox1* (prospero homeobox 1) and *Trim32* (tripartite motif containing 32). These are believed to be translationally repressed in the dividing RGC because the Prox1 protein is not expressed in the progenitors (left side). Following segregation, the mRNAs, which encode cell fate determinants, are translated and act to promote differentiation and suppress the stem cell state (right side). It has not yet been determined whether Stau2 remains associated with the transcripts in neurons during translation or is removed. **(B)** Model for Stau2 function during group 1 metabotropic glutamate receptor (mGluR) long-term depression (LTD). Stau2 RNPs are localised near synapses. Stau2 interacts and colocalises with the nuclear cap-binding protein (CBP80) and the translational repressors FMRP (fragile X mental retardation protein) and Pum2 in dendrites of mature neurons. In addition, Stau2 interacts with components of miRISC (miRNA-induced silencing complex) in the brain. Signalling through mGluRs leads to translation of localised mRNAs, which encode so-called ‘LTD proteins’. These contribute to the endocytosis of AMPA receptors (AMPARs), resulting in depression of the synapse. The complement of mRNAs required for LTD has not yet been determined. We propose here that Stau2 RNPs are disassembled/remodelled following synaptic stimulation, allowing translation of target mRNAs, such as *Rgs4* and *Map1b* (microtubule associated protein 1B), which contribute to the modification of the ‘activated’ synapse. FMRP knockout mice exhibit enhanced mGluR-LTD, whereas Stau2 knockdown impairs LTD, therefore the two proteins may have antagonistic effects.

If Stau2 were responsible for segregating cell fate determinants then, in its absence, both daughter cells should receive those factors, which would promote differentiation and suppress the stem cell state. What then are these putative cell fate determinants? Interestingly, the mRNA encoding the homologue of *Drosophila pros, prospero homeobox 1* (Prox1), was also associated with Stau2 in the mouse brain as it is in the fly (also Box 1). Together with two other common ribonucleoprotein particle (RNP) components, Pum2 and Ddx1, the Stau2/*Prox1* complex is asymmetrically distributed [6]. Pum2 is also associated with Stau2 in mature neurons and colocalises with Stau2 in dendrites of mature hippocampal neurons [9]. This suggests that it too represents another conserved component of the RNA localisation machinery in both neurogenesis and synaptic plasticity (Figure 2). Kusek *et al.* (2012) additionally found that Stau2 affects the asymmetric distribution of *Trim32* and *Bbs2* mRNA [7]. Trim32 is the mouse homologue of *Drosophila* Brat, whose protein also asymmetrically localises in fly neuroblasts [10]. Furthermore, Trim32 has previously been shown to regulate neurogenesis in mice [11]. In progenitor cells, the apically localised *Prox1* mRNA is translationally repressed because Prox1 protein is absent from these cells [6]. The authors therefore propose that upon asymmetric cell division repression is relieved allowing expression of Prox1 and other cell fate determinants (Figure 2A).

In summary, it is fair to conclude that Stau2 is important for distributing cell fate determinants that then suppress the stem cell state and promote differentiation. The findings that Stau2 regulates *Prox1* and *Trim32* (Brat homologue) suggest that specific mRNAs may be conserved between fly and mouse neurogenesis. These transcription factors promote neurogenesis via different mechanisms. The former is via relief of notch1 inhibition on neurogenesis [12], whereas the latter is partly via the enhancement of the activity of several microRNAs [11]. Furthermore, Kusek *et al.* found that Stau2 associates with multiple mRNAs encoding components related to cilia function and signaling. Regulation of these transcripts could lead to the differential response of daughter cells to extracellular ligands, and this is another known means to achieve different cell fates during asymmetric cell division [7]. Therefore, different mechanisms are likely to act in concert to promote neuronal cell fate – where one common link is the localisation of functionally related mRNAs via Stau.

## Stau proteins in synaptic plasticity and memory formation

A growing suite of evidence now shows that Stau proteins are not only important for neurogenesis during early development but that they also play another important functional role in the mature nervous system. Data from *Drosophila*, *Aplysia*, and mouse all indicate a conserved role in dendrite development, synapse function, and plasticity (Box 1).

In the fly, Stau is required in the adult brain during long-term memory (LTM) formation 13, 14, 15, 16, 17. *Stau* expression is induced under conditions of LTM formation via the NMDA receptor, which leads to its CREB (cAMP response element binding protein)-dependent transcription 13, 16. Not only is one-day memory abolished in temperature-sensitive *stau* mutants [13], but a genetic interaction between FMRP (fragile X mental retardation protein) and Stau in the formation of one-day memory in double heterozygote flies implies the role of these two RBPs in this process is related [14]. A recent study now also demonstrates the importance of *stau* in the activity-dependent structural plasticity of *Drosophila* larval motor neuron dendrites [17]. In this case, neuronal activity leads to the CaMKII (Ca^2+^/calmodulin-dependent protein kinase II)-dependent phosphorylation of the transcription factor Adf1 (Adh transcription factor 1), which then negatively regulates the expression of *stau* in motor neurons. In the context of Adf1 induction, Stau acts as a negative regulator of dendritic growth. However, it should be noted that both knockdown and overexpression of Stau reduced dendritic outgrowth of larval motor neurons, suggesting a more complex relationship between Stau and dendritic outgrowth [17].

In rodent primary hippocampal neurons, Stau2 contributes to dendritic spine morphogenesis, the sites of excitatory synapses (Box 1) [18]. In addition, in older neurons Stau2 is required for metabotropic glutamate receptor (mGluR)-induced protein synthesis-dependent long-term depression (LTD) (Figure 1, Figure 2) [19]. This phenotype is independent of its role in dendritic spine morphogenesis [19]. LTD is a form of synaptic plasticity that is associated with internalisation of AMPA receptors and a weakening of synapses in response to synaptic activity [20].

Although orthologous, Stau1 and Stau2 seem to have non-redundant functions in mature neurons. This is probably due to the targeting of different mRNAs and different mechanisms of action on those targets (discussed below). In contrast to Stau2, knockdown of Stau1 by small interfering RNAs (siRNAs) in hippocampal slice cultures impairs the chemically induced NMDA receptor-dependent late form of long-term potentiation (L-LTP), but not the early form, nor LTD [21]. This type of synaptic plasticity is also transcription- and translation-dependent, but results in a strengthening of synaptic connections. Similarly to Stau2 knockdown neurons Stau1-deficient mice exhibit a decrease in dendritic protrusions, which in turn appear to be elongated, resulting in fewer synapses [22]. Additionally, Stau1 mutant neurons show impaired dendritic outgrowth [22]. Again, the effect of Stau1 on L-LTP is independent of its effect on dendritic spine morphology. However, both the morphological as well as the electrophysiological effect are mediated via the NMDA receptor [23]. Despite the morphological defects of the Stau1 mutant mice in synapse development, only a deficit in locomotor activity has been detected *in vivo*, whereas learning and memory formation appears to be normal [22]. Importantly, Stau2 is not upregulated in the Stau1 mutant mice, arguing against a compensatory mechanism between the two paralogues [22]. However, redundancy or compensation by other genes during development cannot be ruled out, especially given that Stau1 and Stau2 have been reported to heterodimerise, at least in some cell lines [24]. In summary, both proteins are involved in dendritic spine morphogenesis, and this seems to be independent of the effects on plasticity 19, 23. In addition, Stau1 and Stau2 appear to have non-redundant functions in protein synthesis-dependent forms of L-LTP and LTD at hippocampal synapses, respectively.

### What are the molecular functions of Stau proteins in synaptic plasticity?

Together, the aforementioned results demonstrate the importance of Stau proteins for several forms of protein synthesis-dependent synaptic plasticity in different organisms. What is less clear is the underlying molecular causes for these defects in Stau mutants. Is misregulation of individual Stau target mRNAs responsible for the defects in plasticity? Or are many mRNAs misregulated in the absence of Stau1/2 that collectively lead to defects at the synapse?

Several studies have estimated the number of mRNAs associated with Stau2 in the brain or cell lines to be approximately 1200 different mRNAs 7, 25, 26. The number of physiologically relevant targets that are directly regulated by Stau2 is, however, likely to be much smaller [27]. For example, these studies isolated total RNA from Stau2 RNPs, and these may include mRNAs bound by other RBPs within the particle. Furthermore, of the 1200 mRNAs associated with Stau2 in embryonic rat brain, the steady-state levels of only 38 were influenced by Stau2 downregulation in neurons (discussed below) [27]. However, there may be more functionally relevant targets where Stau2 regulates localisation and/or translation but not steady-state mRNA levels, for example.

Studies in rodents indeed suggest roles in localisation that are independent of an effect on mRNA stability. In hippocampal neurons, both Stau proteins form RNPs that traffic in neurons to distal dendrites via microtubules 18, 28. Expression of a dominant-negative Stau2 in neurons reduces total dendritic RNA by 40%, while concomitantly increasing the somatic RNA levels [29]. Consistent with this, the vast majority of Stau2 coprecipitating mRNAs from rat brain are localised in the neuropil layer of the hippocampus – a layer which is dense in neuronal processes and devoid of cell bodies 27, 30.

Interestingly, the identified 38 functional targets were highly enriched for synaptic proteins, suggesting that Stau2 does indeed regulate biologically related mRNAs [27]. Of these, mRNAs encoding proteins with known roles in synaptic plasticity and behaviour were identified, including the regulator of G-protein signaling 4 (*Rgs4*) and complexin 1 (*Cplx1*). In addition, the *Camk2a* (Stau1), microtubule associated protein 1B/*Map1b* (Stau2), and β-actin/*Actb* (Stau2) mRNAs have been implicated in transport by the Stau homologues 18, 19, 31. It is therefore reasonable to hypothesise that misregulation of synaptic proteins encoded by target mRNAs underlies the described Stau protein phenotypes.

mGluR-dependent LTP and LTD both require new protein synthesis. For LTD, the model assumes that, upon stimulation of a particular synapse, mGluR-mediated signaling leads to the derepression and translation of localised mRNAs encoding ‘LTD’ proteins. (Figure 2B) [32]. To date only a handful of LTD proteins have been described, including Map1b. The theory is that they encode proteins that contribute to the endocytosis of AMPA receptors and the depression of the synapse. It is therefore likely that the newly identified Stau2 target mRNAs encoding synaptic proteins represent additional LTD proteins that contribute to this phenomenon.

## Towards an understanding of the molecular machine that guides RNA localisation

In the *Drosophila* oocyte, Stau has been implicated in the transport [33], anchoring 33, 34, 35 and translation activation 36, 37 of the *oskar* mRNA to the posterior pole. It is also crucial for the localisation of the *bicoid* mRNA to the anterior pole of the oocyte [38]. In both cases, once localised, Stau protein stays associated with the mRNA throughout anchoring and translation, suggesting roles in all of these processes [39].

The general model for RNA localisation posits that an mRNA is packaged with proteins into RNPs in the nucleus, and the complex is then exported to the cytoplasm and transported in a translationally silent state to its destination where translation can be activated [1]. RNPs are thought to undergo remodelling because proteins can be added or removed at all points. In neurons, new *in vivo* imaging data indicates that mRNAs and ribosomes are ‘unmasked’ in response to synaptic activity 40, 41. This unmasking refers to increased accessibility to the mRNA by probes, and this is thought to correlate with a degranulation of RNPs that allows increased mRNA translation in response to stimuli 40, 41. Following translation, the localisation process eventually ends with the decay of the mRNA, presumably locally at the synapse. In this context, Stau proteins are likely to contribute to several distinct steps of the localisation process.

### Stau proteins and translational control

Recent work has identified the complement of proteins and RNAs present in Stau2 granules in the brain, yielding better understanding of Stau2 function 9, 26, 27, 31, 42. Our laboratory has used biochemical fractionation of endogenous brain lysates to remove (rough) endoplasmic reticulum (ER)-associated Stau2 from the input material to identify Stau2 interactors from the non-membrane bound pool of Stau2 [9]. In this work, two different neuronal RNPs (Barentsz and Stau2) were compared; this provided evidence that transcripts are translationally repressed during transport. Importantly, a series of known translational repressors are enriched in the Stau2 RNPs, including FMRP, Pura (purine-rich element binding protein A), and DDX6 [DEAD (Asp-Glu-Ala-Asp) box helicase 6, also known as Rck], as well as several components of the RNA-induced silencing complex (RISC) [9]. Consistent with these observations, there was no enrichment of translation initiation factors. Further supporting translational repression is the findings that the nuclear proteins CBP80 (cap binding protein 80) and PABPN1 (polyadenylate binding protein 1, nuclear) were both identified in the Stau2 RNPs [9]. First, this provides evidence that the complexes are initially assembled in the nucleus. Second, the presence of CBP80 and PABPN1, together with the absence of eIF4E (eukaryotic translation initiation factor 4E), suggest that translation is stalled because they are normally replaced by eIF4E once steady-state translation is established [43]. Interestingly, however, given that the exon junction complex (EJC) seems to be largely absent from Stau2 RNPs, it may be that the mRNAs have undergone a first round of translation [9]. On the other hand, there is some evidence that the EJC component eIF4AIII is present in Stau2 particles, raising the possibility that it is involved in an EJC-independent function in this case [9]. In this context, eIF4AIII has previously been shown to regulate the expression of the *Arc* (activity regulated cytoskeletal-associated protein) mRNA in dendrites, leading to changes in synaptic strength [44]. Likewise, the presence of CBP80 and PABPN1 may be independent of their known role in the initial round of translation. It should also be noted that although CBP80 and PABPN1 are present in Stau2 granules, it remains to be shown directly that all proteins are indeed present on an individual mRNA. However, CBP80 and Stau2 do partially colocalise in distal dendrites of hippocampal neurons [9].

As mentioned above, an association of Stau proteins with the rough ER and ribosomes has been well documented, indicating a link to translation 45, 46, 47, 48. Biochemical fractionation and immunofluorescence of Stau2 in hippocampal neurons suggests that there might be (at least) two pools of the protein: one associated with the rough ER that is part of large complexes and mostly immobile, and a soluble fraction in smaller mobile RNPs that are found in distal dendrites 45, 49. The larger complexes cofractionate with ribosomal and ER markers, whereas the smaller complexes do not. Consistent with a role in translation, it was recently shown that Stau1 associates with actively translating ribosomes in human cells [48]. These findings fit with previous publications that link Stau1 to ribosomes and translation 50, 51, 52.

It remains to be seen how Stau targets are translationally regulated once localised. The use of high-resolution imaging has recently shed some light on this area. Experiments in which endogenous mRNAs were labelled and tracked following depolarisation of neurons indicate that RNP granules disassemble in response to activity, allowing translation to proceed 40, 41. In the future, it will be interesting to use such imaging techniques to see how Stau proteins are regulated during neuronal activity and whether they influence granule assembly or translational control.

### Stau proteins and mRNA stability

In cell lines, mammalian Stau1 has been reported to target mRNAs for degradation via a process termed Staufen-mediated decay (SMD) 53, 54. SMD is a translation-dependent decay pathway where Stau1 binds to target 3′-untranslated regions (UTRs) and recruits the helicase up-frameshift 1 (Upf-1) to elicit mRNA decay [55]. Stau2 was also recently implicated in SMD in human cells [24]. Physiologically, SMD contributes to the differentiation of myoblasts, the motility of keratinocytes and the differentiation of adipocytes 56, 57, 58.

We recently reported the impact of Stau2 downregulation on target mRNAs in primary cortical neurons [27]. Interestingly, 32 of the associated mRNAs were downregulated, whereas six were upregulated following Stau2 knockdown. This suggests that Stau2 preferentially mediates the stabilisation of target mRNAs rather than their destabilisation [27]. Several new studies also support the findings from neurons, showing Stau-mediated stabilisation in HEK293F cells, neuroblastoma cells and myoblasts 59, 60, 61. In one notable example, the long non-coding RNA (lncRNA) terminal differentiation-induced ncRNA (TINCR) guides Stau1 to target mRNAs through a complementary 25 nt sequence in the lncRNA and mRNA (Box 2) [62]. This association leads to the stabilisation of the mRNA. Such an elegant mechanism would provide a way to direct Stau proteins to subsets of mRNAs in different tissues through regulated expression of specific lncRNAs.

Box 2 **Stau-recognised structures (SRS)**

Stau proteins belong to the large family of dsRBPs which bind specifically to double-stranded RNA (dsRNA; as opposed to dsDNA or single-stranded nucleic acids) via dsRNA-binding domains (dsRBDs) [85]. New studies detail the numerous possibilities for the formation of secondary structures that are recognised by Stau proteins.

Figure IA*Intramolecular structures formed in cis within an mRNA molecule.*

• The 3′-UTR of the SMD target, ARF1 (ADP-ribosylation factor 1), requires a 19 bp stem-loop for regulation by Stau1; however, similar structures could not be found in other SMD targets [53].
• A new study found that the open reading frames (ORFs) and 3′-UTRs of Stau1 targets have a high overall secondary structure or high GC content in human cell lines [48].
• In addition, Stau1 targets are enriched for two inverted *Alu* elements which can fold to form a long dsRNA structure. This is recognized by Stau1 and can enhance their nucleocytoplasmic export 48, 86, 87.
• In *Drosophila*, genome-wide screening identified three dsRNA structures that were enriched in Stau targets [75]. The double-stranded regions were formed in *cis*, and have only a small number of mismatched bases, zero or few unpaired bases, and short internal loops. One of these structures appears to be conserved in mammals because it was also enriched in rodent Stau2-stabilised target mRNAs [27].

**Figure I:**
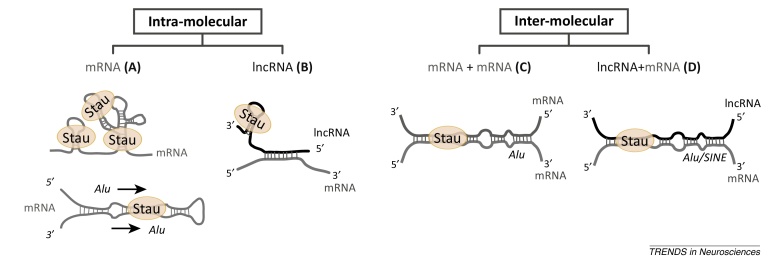
Diversity of Staufen-recognised structures.

Figure IB *Formation of intramolecular structures within long non-coding RNAs (lncRNAs)*.

• Recently, the TINCR lncRNA was found to associate with Stau1 in differentiating keratinocytes [62]. An independent region of TINCR base-pairs with complementary mRNAs, thus recruiting Stau1 to the mRNA to mediate its stabilisation. The structure within the lncRNA was not yet precisely defined.

Figure IC *Intermolecular base-pairing between the 3′-UTRs of two mRNAs.*

• *Alu* elements within the 3′-UTRs of two mRNAs can additionally form double-stranded structures that are recognized by Stau1 in human cells. This can result in the degradation of both mRNAs via SMD, provided that both are translated [88].

Figure ID *Intermolecular base-pairing between a lncRNA and an mRNA.*

• A Stau1 SRS can be formed when highly complementary short interspersed elements (SINEs, e.g., *Alu*) within a lncRNA and an mRNA interact to produce long dsRNA structures 56, 89. The interaction of the two RNAs and binding of Stau1 results in decay of the mRNA via SMD.

Together, these studies suggest that cell type-specific differences exist with regard to the effect of Stau proteins on target mRNA stability. Stau1 and Stau2 interact directly with Upf1 in an RNA-independent manner in human cells [24]. By contrast, in embryonic rat brain this interaction appears to be RNA-dependent [9], providing one possible explanation for the different effects on mRNA stability between different cell types, given that Upf1 is crucial for SMD. On the other hand, a new study which investigated the global profile of Stau1 binding in human cells and the impact of Stau1 downregulation could find little evidence for destabilisation of target mRNAs, which would be expected for SMD [48]. In conclusion, these studies highlight the need for further investigation into the impact of Stau proteins on mRNA stability to determine how both stabilisation and destabilisation might be controlled and under what physiological conditions these regulatory events occur.

### Are Stau proteins important for RNP assembly?

New advances into how non membrane-bound RNP granules assemble suggest that the Stau proteins may be involved. Several new papers from the McKnight laboratory provide compelling evidence that low complexity (LC) polypeptide sequences can reversibly polymerise into uniform amyloid-like fibres to form the structural basis for many types of cellular RNA granules, such as neuronal transport granules, stress granules, and P-bodies [63]. LC sequences are protein domains composed of amino acids with very little diversity. These reports extend previous studies showing that LC sequences are important for localising RBPs to P-bodies in yeast and to stress granules in mammalian cells 64, 65, 66. The authors propose that LC sequences, which are especially enriched in nucleic acid-binding proteins, can exist in one of three states: (i) soluble proteins, (ii) dynamic and reversible polymerised fibres, and (iii) irreversible insoluble aggregates (as seen in some pathologies [63]). Interestingly, Stau1 was one of the 106 RBPs precipitated with the granules from all four different tissues or cell types tested (NIH-3T3, mouse ES cells, mouse brain, and mouse testis) [63]. This suggests that Stau proteins contain LC sequences that polymerise to form granules in many different cell and tissue types, validating its classification as an important RNP component. Rewardingly, the mRNA components of Stau2 granules identified in rat brain [27] significantly overlap with mRNAs precipitated in the LC-domain granules from mouse brain [67], arguing that they are the same RNPs.

The formation of LC-domain fibres is dependent on a high local concentration of protein, and can represent homo- or heterotypic fibres consisting of one or many LC-containing proteins. It is likely that, *in vivo*, RNA sequences provide the scaffold to create a high local concentration of the RBP to polymerise the LC domains. In a biological sense, this observation would fit with the ‘RNA signature’ model of post-transcriptional regulation [68] where *cis*-elements in an mRNA guide the binding of multiple different RBPs and *trans*-acting factors. In this case, the RNA would provide the local environment to polymerise different sets of proteins into a unique RNA granule to achieve its cellular fate. Whether the LC domains of Stau proteins are indeed important for RNP formation and dynamics is an interesting prospect that warrants further investigation.

## Concluding remarks

Early work established a conserved function of Stau proteins in embryonic development in species ranging from flies to frogs, pigs and zebrafish 69, 70, 71, 72, 73, 74. As discussed here, the conservation of Stau protein functions clearly extend beyond the embryo to neurogenesis and synaptic development and plasticity (Figure 1). It is therefore fair to conclude that Stau is a common factor in the RNA localisation machinery that is utilised by different cell types under different conditions.

This raises many questions about how mRNA target selection changes and how this is regulated (Box 2; further outstanding questions are given in Box 3). In this respect, recent studies have indicated that a thorough approach to identifying targets is necessary 27, 75. The expression level of Stau proteins can have a large impact on how many mRNAs are bound and identified, and even modest overexpression of Stau can lead to the identification of 10-fold more (probably spurious) targets [75]. This observation could be physiologically relevant, however, because expression levels of Stau proteins in different cellular conditions may be regulated to modulate target selection.

Box 3 **Outstanding questions**

*(i)* *Molecular roles of Stau proteins*
  • How is translation regulated in Stau2 RNPs? Are ribosomes associated with the RNPs during transport or only once localised? How is ribosome association regulated?
  • Do Stau proteins regulate the dynamics of neuronal RNA granule assembly/disassembly via their LC domains?
  • Is RNP assembly regulated by post-translational modifications of RBPs, for example phosphorylation, as was suggested for the RBP FUS (fused in sarcoma)?
  • How do Stau proteins regulate both mRNA stabilisation and destabilisation? What are the differences between mRNAs that are targeted for one fate versus the other?
  • Both mammalian Stau homologues are expressed in multiple isoforms. Do different isoforms have distinct or overlapping functions in neurons? How are these functions affected by post-translational modifications?
  • When and where are RNPs formed and how are they remodelled over time? The identification of nuclear proteins in the Stau2 RNPs indicates that these are initially formed in the nucleus. It remains unclear at which stage during RNP assembly different proteins are recruited.
  • How do Stau proteins recognize dsRNA structures of varying lengths? Structural studies looking at the interaction between Stau dsRBDs and natural targets are necessary to determine the requirements of target recognition.
  • What lncRNAs are associated with Stau proteins in different cell types? Do the lncRNAs guide Stau to different target mRNAs as is the case for the TINCR lncRNA in keratinocytes?
*(ii)* *Physiological functions of Stau proteins*
  • How are Stau proteins regulated by neuronal activity? In *Drosophila*, *stau* is transcriptionally induced under conditions of long-term memory formation. Although it has been reported in mammals that Stau1 and Stau2 are important for LTP and LTD, respectively, it has not been determined how the RBPs respond to these induction protocols. How does neuronal activity influence RNP assembly/disassembly?
  • What is the underlying cause of the defects in LTP and LTD seen in Stau1 and Stau2 knockdown, respectively? Can the misregulation of specific mRNAs be linked to these defects? Do Stau proteins truly regulate mRNA expression locally at the synapse in response to synaptic activity?
  • How does the repertoire of Stau2 target mRNAs change during different stages of development? Embryonic RGCs give rise to neurons, whereas postnatal RGCs give rise to various glial populations. Is Stau2 also important for gliagenesis as well as neurogenesis?
  • What is the phenotype of Stau2 knockout mice? Are there defects in learning and memory formation? Are there any effects akin to neurodevelopmental or neuropsychiatric disorders? Can different developmental effects in the brain be distinguished, for example as a result of defects in neurogenesis or defects in the plasticity of mature neurons?

In recent years, coupling of coimmunoprecipitation studies with a second independent method to distinguish direct Stau targets (e.g., the identification of secondary structures in transcripts, or global functional assays such as Stau2 knockdown to identify affected mRNAs) has been most effective in identifying relevant targets. Future screens that are directed at analysing specific steps in the RNA localisation pathway will no doubt uncover new functional target mRNAs. For example, Stau2 may only regulate these additional targets at the level of RNA transport but not stability.

Deciphering the mechanisms underlying post-transcriptional regulatory mechanisms in the brain is crucial for not only understanding normal neuronal function but also the disease state. Mutations that lead to the misregulation of several RBPs underlie many neurologic diseases, including fragile X syndrome (FXS), amyotrophic lateral sclerosis (ALS), spinal cerebellar ataxias (SCA), and Huntington's disease (HD) (reviewed in 76, 77). Stau proteins are known interactors of several of the implicated RBPs, and a clearer picture of their role in the developing and adult brain is very likely to provide novel insights into the disease state.
